# The Pelvic Organ Prolapse/Urinary Incontinence Sexual Questionnaire (PISQ-12): validation of the Dutch version

**DOI:** 10.1007/s00192-015-2692-y

**Published:** 2015-05-12

**Authors:** Lisette A. ‘t Hoen, Elaine Utomo, Anneke B. Steensma, Bertil F. M. Blok, Ida J. Korfage

**Affiliations:** Department of Urology, Erasmus Medical Center, Room Na-1724, PO Box 2040, 3000 CA Rotterdam, The Netherlands; Department of Obstetrics and Gynecology, Erasmus Medical Center, Rotterdam, The Netherlands; Department of Public Health, Erasmus Medical Center, Rotterdam, The Netherlands

**Keywords:** Sexual dysfunction, Pelvic floor disorders, Urinary incontinence, Translations, Validation, Dutch

## Abstract

**Objectives and hypothesis:**

To establish the reliability and validity of the Dutch version of the Pelvic Organ Prolapse/Urinary Incontinence Sexual Questionnaire (PISQ-12) in women with pelvic floor dysfunction.

**Methods:**

The PISQ-12 was translated into Dutch following a standardized translation process. A group of 124 women involved in a heterosexual relationship who had had symptoms of urinary incontinence, fecal incontinence and/or pelvic organ prolapse for at least 3 months were eligible for inclusion. A reference group was used for assessment of discriminative ability. Data were analyzed for internal consistency, reproducibility, construct validity, responsiveness, and interpretability. An alteration was made to item 12 and was corrected for during the analysis.

**Results:**

The patient group comprised 70 of the 124 eligible women, and the reference group comprised 208 women from a panel representative of the Dutch female population. The Dutch PISQ-12 showed an adequate internal consistency with a Cronbach’s alpha of 0.57 – 0.69, increasing with correction for item 12 to 0.69 – 0.75, for the reference and patient group, respectively. Scores in the patient group were lower (32.6 ± 6.9) than in the reference group (36.3 ± 4.8; *p* = 0.0001), indicating a lower sexual function in the patient group and good discriminative ability. Reproducibility was excellent with an intraclass correlation coefficient for agreement of 0.93 (0.88 – 0.96). A positive correlation was found with the Short Form-12 Health Survey (SF-12) measure representing good criterion validity. Due to the small number of patients who had received treatment at the 6-month follow-up, no significant responsiveness could be established.

**Conclusions:**

This study showed that the Dutch version of the PISQ-12 has good validity and reliability. The PISQ-12 will enable Dutch physicians to evaluate sexual dysfunction in women with pelvic floor disorders.

## Introduction

Women who suffer from pelvic floor disorders (PFD) generally experience a reduced quality of life [1]. PFD includes urinary incontinence (UI), pelvic organ prolapse (POP) and/or fecal incontinence (FI). The prevalence of PFD in adult women has been estimated to be 23.7 % and increases with age up to 49.7 % in women aged 80 years or older [2]. Several studies have shown that women suffering from UI and POP experience a deterioration in sexual function [3–5], and FI has also been associated with poorer sexual function [6]. Given the number of women suffering from PFD, it is important to evaluate their sexual function. Questionnaires can be used to assess the necessity for treatment of sexual dysfunction, and to determine treatment effectiveness.

The Pelvic Organ Prolapse/Urinary Incontinence Sexual Questionnaire (PISQ-12) is a validated condition-specific quality of life questionnaire [7]. The International Continence Society recommends this questionnaire (with grade A) to assess sexual function with urinary symptoms [8]. In recent years the PISQ-12 has been validated in different languages: Arabic, Chinese, French, Persian, Portuguese, Swedish and Turkish [9–15]. The increased awareness of sexual dysfunction in association with PFD strengthens the need for a validated Dutch measurement tool for sexual function [16]. A recent study has used a Dutch translation of the PISQ-12 to evaluate sexual function in the Dutch population [17]. However, this version was not validated and the results can therefore not be considered internationally compatible. The aim of this study is to develop a validated Dutch version of the PISQ-12 measure.

## Materials and methods

This observational study was performed at a tertiary pelvic floor center. It is part of a larger health-related quality of life study, which was approved by the medical research ethics committee (MEC-2008-376) [18, 19].

### Study populations

#### Patient group

Women were eligible for inclusion if they spoke Dutch fluently, were aged over 18 years, and in a heterosexual relationship. Also, they needed to have been experiencing symptoms of UI, POP stage 2 or higher and/or FI for at least 3 months. Exclusion criteria consisted of dementia, mental retardation, active malignant tumors, and no sexual activity during the past 6 months. During a regular outpatient visit all potentially eligible patients were informed about the study, and invited to participate by their treating physician. A patient information package containing the consent form and the first two sets of measures were handed out. Patients were asked to fill in the questionnaires at three predetermined time-points (during the inclusion visit, and at 1 week and 6 months after inclusion), and to return the questionnaires by post directly after completion. The patients’ educational levels were determined and classified as “lower” (primary school), “middle” (high school), and “higher” (college or university). The final questionnaire contained an extra question taken from the RAND 36-Item Health Survey (RAND 36-HTI). Patients were asked to compare their current general health status to their status 1 year ago [20]. No treatment was initiated during the first week following inclusion. Physicians and patients were unrestricted in the choice of treatment given the observational character of this study. For treatment evaluation we distinguished conservative, pharmaceutical and surgical treatments.

#### Reference group

The reference group in this study was taken from an ISO-certified (ISO 26362) panel of Dutch women 18 years of age or older. This group was stratified for age, educational level and residence to act as a representative group of the Dutch female population. Beforehand, the presence of pelvic floor symptoms in this group was unknown.

### Questionnaire

The questionnaire consisted of two measures:

- The PISQ-12 is a short-form of the PISQ-31 measure [21]. It is a condition-specific measure that evaluates sexual function in heterosexual women who suffer from UI and/or POP. The PISQ-12 measures three domains: behavioral-emotive (items 1 – 4), physical (items 5 – 9) and partner-related (items 10 – 12). It is a self-administered questionnaire, and responses are graded on a five-point Likert scale ranging from 0 (always) to 4 (never). Items 1 – 4 are reversely scored and a total of 48 is the maximum score [22]; higher scores indicate better sexual function. Up to two missing responses are accepted. The total score sum with missing values is calculated by multiplying the number of items by the mean of the responses to the items reported by that person. The PISQ-12 is reported as a single sexual function score. It does not report the separate domains [7].
- The Short Form-12 Health Survey (SF-12) consists of two summary measures, physical component scores (PCS-12) and mental component scores (MCS-12) [23]. It is the short-form of the SF-36 measure and is frequently used as a gold standard for health-related quality of life questionnaires [14, 10]. The SF-12 was distributed to the patient group only.

#### Linguistic validation

The translation process of the PISQ-12 was performed according to a standardized guideline [24]. First the English PISQ-12 was forward-translated by three independent native Dutch speakers. Differences were discussed and consensus was reached on the final version, which was then backward-translated by a native English speaker. A face-to-face test with ten patients was performed, and small textural changes were made accordingly without the need to adapt the content, resulting in the final Dutch version (Appendix).

### Measurement properties

The questionnaire was validated according to the following measurement properties;

1. Content validity: the extent to which the questionnaire measures the concepts of interest in the target population. The correspondence between the questionnaire items and clinical symptoms was subjectively assessed by the researchers. Face validity was determined by the researchers and a selected group of patients during linguistic validation [25].
2. Internal consistency: the correlation between different items in a questionnaire for the total and subscale scores, i.e. do the questions measure the same construct? A Cronbach’s alpha was calculated for the total score and three subscale scores separately. Internal consistency is considered good if the Cronbach’s alpha was between 0.70 and 0.95 [25]. For study-related reasons we also calculated the Cronbach’s alpha of the PISQ-12 minus item 12 for total score and the partner-related subscale score.
3. Reproducibility: the degree to which scores on a questionnaire are similar in a stable person on repeated measurements. This can be reported through reliability and agreement [25].
  1. Reliability considers the degree to which patients can be differentiated from each other, despite measurement error. The intraclass correlation coefficient (ICC) for agreement was calculated to assess the test–retest reliability. A value of at least 0.70 is the minimum standard [26].
  2. Agreement concerns the similarity in scores when measured on separate occasions, i.e. the measurement error. The limits of agreement (LOA) were reported and equal the mean change in scores of repeated measurements ± 1.96 × standard deviation (SD) of the changes [27].
4. Criterion validity: the degree to which questionnaire scores correlate with a gold standard. For the PISQ-12 no perfect gold standard exists. Therefore, criterion validity was determined using the SF-12, a quality of life measure resulting in physical and mental summary scores, which was also used in the Chinese validation [10]. Spearman’s correlation was determined with values ranging from −1 to +1. A stronger negative or positive correlation is found when values are close to the extremes [25].
5. Construct validity: the extent to which hypotheses about the scores of a questionnaire in relation to other measures are valid. If at least 75 % of the predefined hypotheses are correct, construct validity is considered adequate [25].
  The predetermined hypotheses were:
  1. Women with lower scores on the PCS-12 have lower scores on the PISQ-12.
  2. Women in the patient group will have lower PISQ-12 scores than women in the reference group.
  3. PISQ-12 scores will increase after women have received treatment.
6. Responsiveness: the extent to which a questionnaire is able to detect clinically important changes over time. This was calculated for all patients who had received treatment. The RAND 36-HTI was used as an external criterion in the determination of the area under the receiver operating characteristic (ROC) curve (AUC) for the PISQ-12 measurements. The AUC shows the ability of a questionnaire to distinguish patients who have improved. An AUC of at least 0.50 is considered adequate [25].
7. Interpretability: the degree to which a qualitative meaning can be assigned to the quantitative questionnaire scores. The minimal important change (MIC) is the minimal change required to indicate a true clinically relevant improvement. The LOA should be smaller than the MIC [25]. The anchor-based ROC approach was used to determine the MIC. The MIC is the optimal ROC cut-off point and is defined as the value for which the sum of the proportions for misclassifications ((1 − sensitivity) + (1 − specificity)) is smallest [28].
8. Floor and ceiling effects: if more than 15 % of the women have received the lowest or highest possible score [25]. Floor and ceiling effects were assessed at the total and subscale levels.

### Statistical methods

To determine the sample size the quality criteria proposed by Terwee et al. [25] were followed. These state that a sample size of at least 50 patients is considered adequate to validate the questionnaire in Dutch. For continuous data we report the mean and standard deviation (SD). For categorical data we report counts and percentages. To evaluate the differences between the patient and reference group Student’s *t* test and the chi-squared test were used for continuous and categorical variables, respectively. One-way analysis of variance (ANOVA) was used for the evaluation of more than two independent groups. A two-sided *p* value of <0.05 was considered significant. The anchor RAND 36-HTI was dichotomized to “improved” and “not improved”; “a little better” and “much better” were classified as “improved”, while “same”, “a little worse” and “much worse” were classified as “not improved”. Statistical analysis of the data was done using SPSS version 21.0 (IBM Corp., Armonk, NY).

## Results

**Fig. 1:**
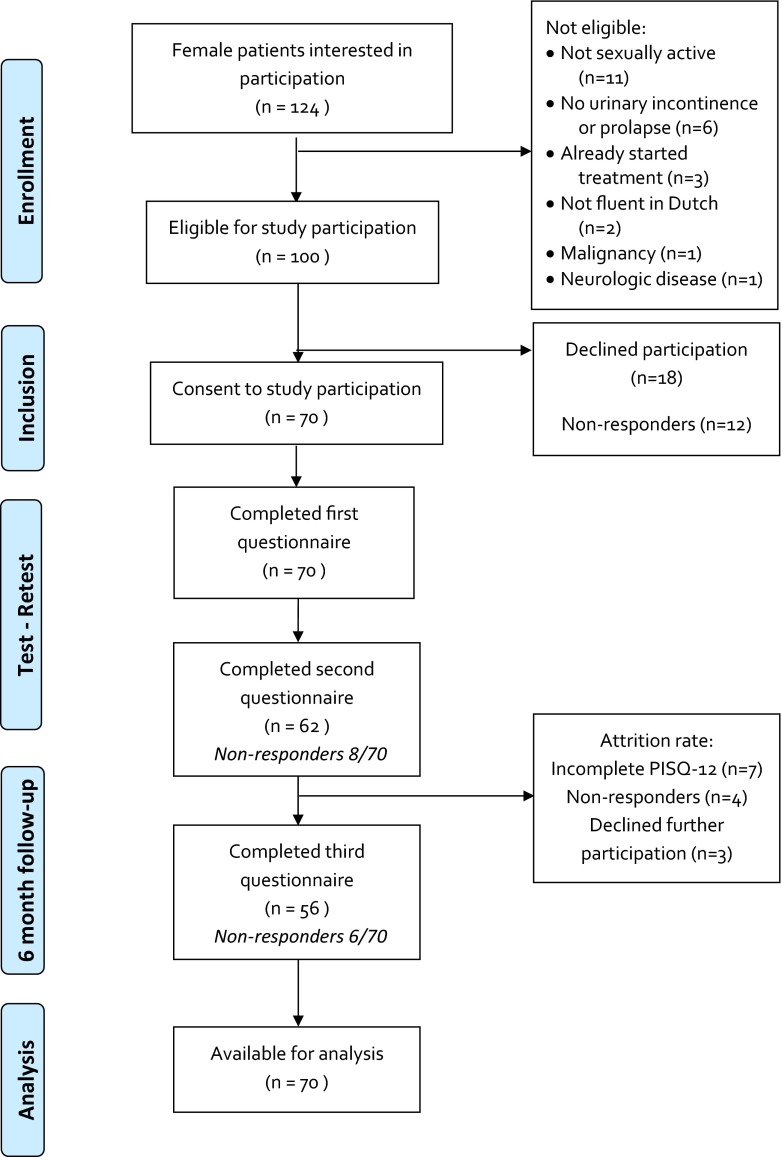
Flow chart showing the inclusion process of the patient group

Of the 124 consecutive patients who were interested in participation in the study, 100 were found to be eligible and 70 of these patients consented to participate after receiving further information. During analysis, data from 70 patients were available, and consisted of at least one completed measure at one time-point (Fig. 1). The reference group consisted of 208 women who had responded of 450 Dutch women contacted. Table 1 displays the nature of PFD in the patient group. The women in the reference group were younger than the women in the patient group (45.1 ± 14.2 years and 53.6 ± 12.3 years, respectively; *p* < 0.001). There was also a significant difference in educational level, with an average higher level of education in the reference group (*p* = 0.004).

**Table 1:** Demographic and clinical characteristics of the study population (patient and reference groups)

	Patient group (*n* = 70^a^)	Reference group (*n* = 208)	*p* value
Age (years), mean ± SD	53.6 ± 12.3	45.1 ± 14.2	<0.001^d^
Education, *n* (%)				
Lower	21 (31)	60 (29)	0.004^e^
Middle	38 (57)	86 (41)	
Higher	8 (12)	62 (30)	
Type of PFD, *n* (%)				
UI	60 (86)		
POP	24 (34)		
FI	17 (24)		
Treatment, *n* (%)				
Conservative	11 (31)		
Pharmaceutical	3 (8)		
Surgical	21 (61)		
PISQ-12 scores, mean ± SD^b^				
Baseline	32.7 ± 7.0	36.3 ± 4.7	0.001^d^
Baseline, minus item 12	30.7 ± 7.1	34.8 ± 5.1	0.01^d^
6 months (*n* = 56)	33.5 ± 6.5		
6 months, minus item 12	31.4 ± 7.0		
SF-12 scores, mean ± SD^c^				
Baseline (*n* = 56)				
PCS-12	41.7 ± 12.0		
MCS-12	48.1 ± 10.5		
6 months (*n* = 46)				
PCS-12	44.6 ± 10.9		
MCS-12	45.4 ± 12.0		

^a^Unless stated otherwise

^b^Higher scores indicate better sexual function

^c^Every score higher than 50 indicates better quality of life, every score lower than 50 indicates poorer quality of life

^d^Student’s *t* test

^e^Chi-squared test

During the layout process of the PISQ-12 the answers to item 12 were incorrectly altered from “much less intense to much more intense” to “always to never”. This unfortunate alteration resulted in suboptimal answer options. Therefore, we calculated the total score without item 12 as well. The reference group showed significant an overall higher scores on the PISQ-12 measure than the patient group, indicating better sexual function in this group. After correcting for the altered item 12, the difference between the patient and reference group remained significant (Table 1).

### Measurement properties

1. Content validity: The content validity was determined to be adequate by the researchers and the selected patient group during the linguistic validation process.
2. Internal consistency: Table 2 shows that the PISQ-12 total score had an adequate internal consistency in the patient group, with a Cronbach’s alpha of 0.69. In the reference group the internal consistency showed a moderate Cronbach’s alpha of 0.57. The domain scales differed between the patient and reference groups from 0.37 – 0.85 and 0.13 – 0.72, respectively. In addition, we calculated the Cronbach’s alpha without item 12. The PISQ-12 showed an adequate internal consistency of 0.75 and 0.69 for the patient and reference groups, respectively. For the partner-related scale internal consistency improved from 0.37 to 0.55 for the patient group, and from 0.13 to 0.49 for the reference group.

**Table 2:** Internal consistency and reproducibility. Cronbach’s alpha reflects the internal consistency for the total and subscale scores. The reproducibility is presented in terms of intraclass correlation coefficient and limits of agreement

	Internal consistency		Reproducibility (*n* = 62)				
	Cronbach’s alpha		Test score (mean ± SD)	Retest score (mean ± SD)	Intraclass correlation coefficient (95 % CI)	Change (mean ± SD)	Limits of agreement^a^
	Patient group	Reference group					
PISQ-12 total	0.69	0.57	32.6 ± 7.1	33.0 ± 7.1	0.93 (0.88 – 0.96)	−0.32 ± 2.71	−5.63 to 4.99
Minus item 12	0.75	0.69	30.7 ± 7.3	31.0 ± 7.3	0.94 (0.90 – 0.96)	−0.32 ± 2.52	−5.26 to 4.62
Behavioral emotive	0.85	0.72	9.58 ± 3.86	9.68 ± 3.69	0.90 (0.84 – 0.94)	−0.05 ± 1.45	−2.89 to 2.79
Physical	0.71	0.62	14.48 ± 4.53	14.71 ± 4.38	0.94 (0.90 – 0.96)	−0.23 ± 1.54	−3.25 to 2.79
Partner-related	0.37	0.13	8.58 ± 2.27	8.63 ± 2.31	0.80 (0.69 – 0.87)	−0.05 ± 1.67	−3.32 to 3.22
Minus item 12	0.55	0.49	6.66 ± 1.80	6.71 ± 2.00	0.85 (0.77 – 0.91)	−0.05 ± 1.03	−2.07 to 1.97

^a^Calculated as: *y* = mean(change) ± 1.96 × SD(change)
3. Reproducibility: A total of 62 patients completed the questionnaires at baseline and the retest after a week (Table 2). The average test–retest period was 6.8 ± 2.6 days. For the PISQ-12 ICC for agreement was 0.93 (range 0.88 – 0.96). This indicates adequate reliability. The ICC for agreement remained adequate after correction for item 12: 0.94 (range 0.90 – 0.96). Relating the LOA range (10.6) to the total PISQ-12 score range (48) resulted in an expected measurement error of 22 %.
4. Criterion validity: The PISQ-12 scores showed positive correlations with the two summary scores of the SF-12 (PCS-12 and MCS-12; Fig. 2). For the PISQ-12 and PCS-12 adequate agreement was found at baseline and the 6-month follow-up (Spearman’s rho 0.41 and 0.34, respectively). The PISQ-12 and MCS-12 showed only adequate agreement at baseline (Spearman’s rho 0.32).

**Fig. 2:**
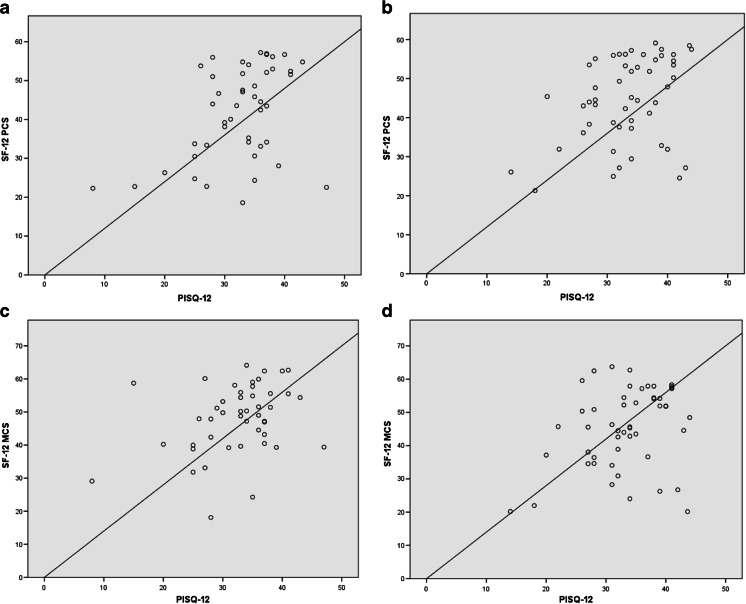
Correlations between and PISQ-12 SF-12 scores to establish the criterion validity. **a** PISQ-12 vs. PCS-12 at baseline (*n* = 56; Spearman’s rho 0.41; *p* = 0.005). **b** PISQ-12 vs. PCS-12 at 6 months (*n* = 46; Spearman’s rho 0.34; *p* = 0.02). **c** PISQ-12 vs. MCS-12 at baseline (*n* = 56; Spearman’s rho 0.32; *p* = 0.03). **d** PISQ-12 vs. MCS-12 at 6 months (*n* = 46; Spearman’s rho 0.26; *p* = 0.07)
5. Construct validity: two of our three predefined hypotheses were confirmed:
  1. A significant correlation was found between the PCS-12 score and the PISQ-12 score (Fig. 2).
  2. Women in the patient group did indeed have lower PISQ-12 scores than women in the reference group (Table 1).
  3. Women who received treatment did not show a significant improvement in PISQ-12 score compared to before treatment (Table 3).

**Table 3:** PISQ-12 scores in patients who received treatment and their corresponding RAND-36 response reflect responsiveness and interpretability of the PISQ-12. The RAND-36 functions as an anchor

	Number (%) (*n* = 24^a^)	PISQ-12 score^b^
RAND-36 health transition item		
Much worse/a little worse	5 (21)	2.60 ± 8.38
Same	10 (42)	−0.10 ± 5.97
A little better	5 (21)	2.20 ± 3.27
Much better	4 (17)	5.00 ± 4.97
Area under the ROC curve		0.69
p-value		0.14
Minimal important change		−0.50
Sensitivity		0.89
Specificity		0.60

Data presented are number (%) or mean change ± SD change between baseline and 6-month follow-up

^a^Responsiveness reported only for the 24 patients who received treatment

^b^Positive scores indicate an improvement in sexual function
6. Responsiveness: At the 6-month follow-up, 56 patients completed the third round of questionnaires as shown in Table 1. Of these patients, 27 had received treatment for either UI, POP or FI. This treatment consisted of surgical treatment in 15 patients (56 %), conservative treatment in 9 patients (33 %) and pharmaceutical treatment in 3 patients (11 %). Of these 27 patients, 24 answered the RAND 36-HTI question. The AUC for the PISQ-12 in this group was 0.69 (*p* = 0.14, not significant; Table 3).
7. Interpretability: The MIC was −0.50 with a sensitivity of 0.89 and a specificity of 0.60 (Table 3). This corresponds with 89 % correctly identified as improved and 60 % as not improved. The MIC was inside the range of the LOA, indicating that the change in score of −0.50 in treated patients who have reported an improvement on the RAND 36-HTI was not clinically relevant.
8. Floor and ceiling effects: The distribution of floor and ceiling effects for the patient and reference groups is shown in Table 4. No floor and ceiling effects were reported in the patient group or the reference group for the PISQ-12 total score. Furthermore, no floor and ceiling effects were reported in the patient group on the scale level. In the reference group, only a ceiling effect on the physical scale was reported.

**Table 4:** Floor and ceiling effects at baseline for the patient and reference groups

PISQ-12	Patient group (*n* = 70)		Reference group (*n* = 208)	
	Floor	Ceiling	Floor	Ceiling
Total score	0 (0 %)	0 (0 %)	0 (0 %)	0 (0 %)
Behavioral emotive	3 (4 %)	0 (0 %)	1 (0.5 %)	2 (1 %)
Physical	0 (0 %)	10 (14 %)	0 (0 %)	53 (25 %)
Partner-related	0 (0 %)	7 (10 %)	0 (0 %)	13 (6 %)

## Discussion

In response to the growing need for a validated measure for sexual dysfunction in women with PFD, the aim of this study was to provide a validated Dutch version of the PISQ-12 measure. In women with PFD significantly lower PISQ-12 scores were observed than in the reference group representing the Dutch female population. This indicates poorer sexual function in the patient group, as expected, confirming the discriminative ability of the PISQ-12 measure. It also stresses the need to consider treatment options for sexual dysfunction in addition to treatment options for PFD alone.

The study showed that the Dutch version of the PISQ-12 has moderate internal consistency. Cronbach’s alpha was 0.69 in the patient group and 0.57 in the reference group. Other studies validating the PISQ-12 in different languages have shown higher Cronbach’s alpha values, ranging from 0.71 to 0.79 [15, 13, 14, 10]. The lower value found in this study originated from the partner-related item scale, probably due to the alteration of item 12. A Cronbach’s alpha for the partner-related scale of 0.37 was found in the patient group, in contrast to 0.13 in the reference group when no adjustment for item 12 was performed. With correction for item 12, Cronbach’s alpha scores for both the total score and the partner-related score increased to values comparable to those found for the earlier translated PISQ-12 versions. The internal consistency of the PISQ-12 then showed an adequate value of 0.75 for the patient group and 0.69 for the reference group. The big discrepancy found between the patient and reference group at the scale level was also resolved with the correction. This suggests that the incorrect answer options for item 12 caused the lower values before correction. Overall, the PISQ-12 showed an adequate consistency for the remaining 11 items.

The internal consistency of the partner-related scale was lower than that of the total score and both the behavioral-emotive scale and the physical scale, even after correction for item 12. This was also found in previous translations of the PISQ-12 [15, 10]. It should be noted that the partner-related scale evaluates the physical sexual function related to the partner. However, even though sexual function is related to the partner, a poorer physical sexual function in the partner will not automatically result in a similar sexual dysfunction in the woman. A lower partner-related scale score does not need to be correlated with the behavioral-emotive and physical scale scores.

The reproducibility for the Dutch PISQ-12 in terms of the test–retest scores was excellent. The ICC for agreement was 0.93, comparable to that of the Swedish PISQ-12 [14], confirming the reproducibility of this measure. With correction for item 12 the reliability remained stable, as expected, since patients received the same version of the measure at both time-points. The good ICC value enables the use of the PISQ-12 as a measure for distinguishing the severity of sexual dysfunction between patients.

The criterion validity of a questionnaire is preferably determined by the degree of its correlation the gold standard. Since no gold standard is available to determine sexual dysfunction in women with PFD we chose to use the SF-12 [23] to assess the criterion validity of the PISQ-12. The SF-12 is a commonly used generic measure for health-related quality of life and was also used in the Chinese validation study of the PISQ-12 [10]. We found a positive correlation between sexual dysfunction as assessed with the PISQ-12 and both summary scores of the SF-12 at baseline. However, at the 6-month follow-up the PISQ-12 showed a significant correlation with the PCS-12, but not with the MCS-12. This weakened correlation at follow-up could potentially be explained by the fact that the group of patients was smaller at 6 months. It is also possible that the SF-12 was not the right choice to assess the criterion validity because the SF-12 is a generic measure: changes in quality of life unrelated to sexual dysfunction might have influenced the SF-12 score without influencing the PISQ-12 score (criterion contamination). We are thus unable to conclude that the PISQ-12 has a good criterion validity using the SF-12. Therefore, we recommend the use of a larger patient group to determine the criterion validity of the PISQ-12.

The responsiveness and interpretability reported were not adequate. Of the 35 patients who did receive treatment during the 6-month follow-up, only 24 completed the third questionnaire including RAND 36-HTI. This small number of patients could explain why the AUC and MIC were not significant. Furthermore, the study was conducted at a tertiary center, where women might present with more severe symptoms of PFD. Treatment options might therefore be more limited, which could explain the only slight overall improvement of PISQ-12 scores after treatment. This probably also contributed to the inability to confirm hypothesis 3 for the construct validity. In addition, the anchor used for responsiveness evaluation, the RAND 36-HTI, solely addresses one aspect of general health, while sexual function is multifactorial. It might therefore not provide an adequate comparison for this specific evaluation.

No floor or ceiling effects were found for the total score of the PISQ-12 for the patient group or the reference group. The complexity of symptoms of PFD could have contributed to the lack of floor and ceiling effects in the patient group. In the reference group ceiling effects were found only on the physical subscale. This has previously also been demonstrated in patients [29]. After treatment the physical subscale showed ceiling effects, while the other subscales did not.

The strength of this study was the use of the quality criteria for the evaluation of the measurement properties tested, as proposed by Terwee et al. [25]. Also, the use of a reference group enabled clarification of differences in sexual function between patients with PFD and a reference population. There are some limitations to this study. First, item 12 was altered during the layout process. In our opinion, errors can (and will) always occur. If an error does occur, we think it best to acknowledge it and try to correct for it in the analysis. Second, the small number of patients during our 6-month follow-up made it difficult to properly assess the responsiveness of the Dutch PISQ-12. Third, the PISQ-12 can only be used in sexually active women. Consequently, an assessment of sexual dysfunction in sexually inactive women cannot be performed. The PISQ-IR is a new questionnaire that does take sexual function and activity or inactivity into account [30]. Therefore, the PISQ-IR can be used in all patients presenting with symptoms of PFD. However, currently no validated Dutch version is available. The actual use of the Dutch PISQ-12 might affect treatment in sexually active women. It would be worthwhile to evaluate the impact of the use of the Dutch PISQ-12 in clinical practice.

In conclusion, this Dutch version of PISQ-12 was tested following well-established guidelines on measurement properties. It was demonstrated to have adequate validity and reproducibility, especially after correction for item 12 on the partner-related scale. The use of this measure in clinical practice will enable Dutch physicians to assess the impact of sexual dysfunction on women with PFD who are sexually active. It is recommended that responsiveness be determined in a larger group of patients.
